# Common genetic variants contribute to heritability of age at onset of schizophrenia

**DOI:** 10.1038/s41398-023-02508-0

**Published:** 2023-06-13

**Authors:** Ester Sada-Fuente, Selena Aranda, Sergi Papiol, Urs Heilbronner, María Dolores Moltó, Eduardo J. Aguilar, Javier González-Peñas, Álvaro Andreu-Bernabeu, Celso Arango, Benedicto Crespo-Facorro, Ana González-Pinto, Lourdes Fañanás, Barbara Arias, Julio Bobes, Javier Costas, Lourdes Martorell, Thomas G. Schulze, Janos L. Kalman, Elisabet Vilella, Gerard Muntané

**Affiliations:** 1 Hospital Universitari Institut Pere Mata, Institut d’Investigació Sanitària Pere Virgili (IISPV), Department of Psychiatry, Universitat Rovira i Virgili (URV), Reus, Spain; 2 Centro de Investigación Biomédica en Red de Salud Mental (CIBERSAM), Madrid, Spain; 3 Institute of Psychiatric Phenomics and Genomics (IPPG), University Hospital, Ludwig Maximilian University of Munich, 80336 Munich, Germany; 4 Department of Psychiatry and Psychotherapy, University Hospital, Ludwig Maximilian University of Munich, 80336 Munich, Germany; 5 Department of Genetics, Universitat de Valencia, 46100 Valencia, Spain; 6 Biomedical Research Institute INCLIVA, 46010 Valencia, Spain; 7 Department of Psychiatry, Hospital Clínico Universitario de Valencia, 46010 Valencia, Spain; 8 Faculty of Medicine, Universidad de Valencia, 46010 Valencia, Spain; 9 Department of Child and Adolescent Psychiatry, Institute of Psychiatry and Mental Health, Gregorio Marañón Health Research Institute (IiSGM), Hospital General Universitario Gregorio Marañón, 28007 Madrid, Spain; 10 Faculty of Medicine, Universidad Complutense, 28007 Madrid, Spain; 11 Department of Psychiatry, Universidad de Cantabria, 39005 Santander, Cantabria Spain; 12 Hospital Universitario Marqués de Valdecilla-IDIVAL, 39008 Santander, Cantabria Spain; 13 Department of Psychiatry, University Hospital Virgen del Rocío, Instituto de Biomedicina de Sevilla (IBiS), Sevilla, Spain; 14 Department of Psychiatry, Hospital Universitario Araba, Instituto de Investigación Sanitaria Bioaraba, Universidad del País Vasco, 01009 Vitoria, Spain; 15 Department of Evolutionary Biology, Ecology and Environmental Sciences, Faculty of Biology, Universitat de Barcelona, Institut de Biomedicina de la Universitat de Barcelona (IBUB), 08028 Barcelona, Spain; 16 Faculty of Medicine and Health Sciences - Psychiatry, Universidad de Oviedo, 33006 Oviedo, Spain; 17 Mental Health Services of Principado de Asturias (SESPA), Instituto de Investigación Sanitaria del Principado de Asturias (ISPA), 33011 Oviedo, Spain; 18 Instituto de Neurociencias del Principado de Asturias (INEUROPA), 33003 Oviedo, Spain; 19 Psychiatric Genetics Group, Instituto de Investigación Sanitaria de Santiago de Compostela (IDIS), Servizo Galego de Saúde (SERGAS), Complexo Hospitalario Universitario de Santiago de Compostela (CHUS), 15706 Santiago de Compostela, Spain; 20 Department of Psychiatry and Behavioral Sciences, SUNY Upstate Medical University, Syracuse, NY US; 21 Institut de Biologia Evolutiva (UPF-CSIC), Departament de Medicina i Ciències de la Vida, Universitat Pompeu Fabra, Parc de Recerca Biomèdica de Barcelona, Barcelona, Spain

**Keywords:** Schizophrenia, Genomics

## Abstract

Schizophrenia (SCZ) is a complex disorder that typically arises in late adolescence or early adulthood. Age at onset (AAO) of SCZ is associated with long-term outcomes of the disease. We explored the genetic architecture of AAO with a genome-wide association study (GWAS), heritability, polygenic risk score (PRS), and copy number variant (CNV) analyses in 4 740 subjects of European ancestry. Although no genome-wide significant locus was identified, SNP-based heritability of AAO was estimated to be between 17 and 21%, indicating a moderate contribution of common variants. We also performed cross-trait PRS analyses with a set of mental disorders and identified a negative association between AAO and common variants for SCZ, childhood maltreatment and attention-deficit/hyperactivity disorder. We also investigated the role of copy number variants (CNVs) in AAO and found an association with the length and number of deletions (*P*-value = 0.03), whereas the presence of CNVs previously reported in SCZ was not associated with earlier onset. To our knowledge, this is the largest GWAS of AAO of SCZ to date in individuals from European ancestry, and the first study to determine the involvement of common variants in the heritability of AAO. Finally, we evidenced the role played by higher SCZ load in determining AAO but discarded the role of pathogenic CNVs. Altogether, these results shed light on the genetic architecture of AAO, which needs to be confirmed with larger studies.

## Introduction

Schizophrenia (SCZ) is a complex disorder influenced by an intricate interplay of genetic and environmental factors. SCZ patients show substantial heterogeneity in clinical characteristics such as symptomatology, cognitive ability, course, overall functioning, and age at onset (AAO). AAO has been consistently included among the most important determinants of disease outcome and is widely accepted as a significant clinical and prognostic factor [1, 2]. For instance, an earlier AAO is associated with a higher likelihood of having relatives with SCZ [3, 4], and has been correlated with an increased number of hospitalizations and illness episodes, more frequent negative symptoms, and poorer cognition, overall functioning, and global outcome [5–7]. In general, men have an earlier AAO, usually between 20 and 24 years of age, while in women, the onset occurs between 25 and 35 years of age [8–10]. In addition, women appear to have a secondary peak around menopause, between 50 and 54 years old [7, 11].

The genetic architecture of SCZ is complex. Heritability estimates from twin and population-based studies range between 64 and 81% [12, 13], in which common genetic variants account for a large proportion (24.4%) [14]. There is also evidence that both rare single-nucleotide variants and rare copy number variants (CNVs) contribute to the risk of developing SCZ [15–19]. In fact, individuals with a pathogenic CNV represent more than 2% of the confirmed cases [20]. CNVs are highly penetrant and may cause early-onset forms of developmental delay or autism spectrum disorders. Thus, similarly, it has been suggested that the presence of CNVs may play an important role in the onset of SCZ, although their contribution is still unclear [20].

The heritability of the AAO has been estimated in sibling pairs at 33% [21], indicating a moderate genetic basis. However, in contrast with the vast amount of information on the genetics of SCZ obtained from genome-wide association studies (GWAS) [14, 22, 23], the genetic determinants underlying AAO remain largely unknown. To date, only three GWAS have been performed in relatively small cohorts (<3000 individuals) and none of them identified any genomic loci associated with AAO at genome-wide significance [24–26]. On the other hand, recent GWAS carried out based on the age at onset of both Bipolar Disorder (BD) and Major Depression Disorder (MDD) with larger sample sizes have determined a significant SNP-based heritability and shared genetic risk with other psychiatric disorders [27, 28]. Thus, further studies with larger sample sizes are required to estimate the contribution of common genetic variants to AAO and the heritability they may explain.

Ultimately, identifying and researching the genetic factors that influence the AAO of SCZ may improve our understanding of the development and progression of this disease, provide new targets for therapy, and facilitate the development of personalized therapeutic interventions and preventive measures. In this study, we aimed to explore the genetic architecture of AAO by performing (1) a GWAS meta-analysis of nearly 5000 subjects of European ancestry, (2) heritability estimates based on common genetic variants, (3) polygenic risk score (PRS) analyses with a set of mental traits, and finally 4) an assessment of the influence of known CNVs on AAO.

## Material and methods

### Sample

Four different datasets, two from Europe (CIBERSAM, PsyCourse) and two from USA (GAIN [29], and nonGAIN), were obtained and combined to perform a GWAS meta-analysis on AAO comprising 4740 patients of European ancestry (Table 1). In all datasets, subjects met the variat for SCZ, schizoaffective disorder, schizophreniform disorder, delusional disorder, brief psychotic disorder, or psychotic disorder not otherwise specified, in the Diagnostic and Statistical Manual of Mental Disorders version IV (DSM-IV).

**Table 1:** Description of samples used in the study.

Dataset	Source	*N*	% Females	Mean AAO (SD)	Genotyping chip
CIBERSAM	Seven groups from the Biomedical Research Network in Mental Health (CIBERSAM)	1704	32.98	25.32 (8.84)	Illumina Infinium PsychArray
PsyCourse	The PsyCourse study	499	38.88	26.26 (9.55)	Illumina Infinium PsychArray
GAIN	The Genome-Wide Association Study of Schizophrenia (dbGaP repository study accession: phs000021.v3.p2)	1280	29.92	21.11 (6.77)	Affymetrix Genome-Wide Human SNP Array 6.0
nonGAIN	The Molecular Genetics of Schizophrenia—nonGAIN Sample (MGS_nonGAIN, dbGaP repository study accession: phs000167.v1.p1)	1224	31.54	21.77 (7.24)	Affymetrix Genome-Wide Human SNP Array 6.0

The CIBERSAM and PsyCourse datasets were collected by the authors, while GAIN and nonGAIN datasets were obtained from public resources. Participants in the CIBERSAM (Biomedical Research Network in Mental Health) dataset were recruited from psychiatric in-patient units at seven different hospitals in Spain [30]. Participation was approved by the ethical committees at the hospitals involved in the recruitment. Finally, samples were genotyped using the Illumina Infinium PsychArray at the Broad Institute as part of the wave 3 meta-analysis GWAS of SCZ of the Psychiatric Genomics Consortium (PGC-SCZ wave 3) [31]. The PsyCourse samples were part of a multi-site German/Austrian longitudinal study (www.psycourse.de) that was conducted between 1 January 2012 and 31 December 2019. The study collected deep phenotypic, neuropsychologic, and omics data from patients with brief psychotic disorder, major depressive disorder (MDD), bipolar disorder (BD), SCZ, schizoaffective disorder, and healthy individuals. Adult participants were referred by the clinical staff or identified by querying patient registries. Study protocols were reviewed and approved by the ethics committees of the Medical Centers and Faculties involved in the recruitment, in accordance with the Declaration of Helsinki. All participants provided written informed consent. The phenotype information was gathered using the v4.1 version of the PsyCourse data release. These samples were genotyped using the Illumina Infinium PsychArray [32]. Finally, GAIN and nonGAIN datasets were both obtained from the dbGaP repository (accession numbers phs000021.v3.p2 and phs000167.v1.p1, respectively), and genotyped with the Affymetrix Genome-Wide Human SNP Array 6.0 as described elsewhere [33].

### Age at onset

In the CIBERSAM dataset, AAO was defined as the onset of the first psychotic symptoms. The patient (and/or family members) and the psychiatrist defined when the first psychological symptoms appeared. In cases where this information was not available, we used the date of the first psychiatric visit due to a psychotic episode. In the PsyCourse dataset, AAO was collected as both the age at first outpatient and inpatient treatment. Subjects with information available for either of these data were included, and when both data were available, the earliest age was used. For the GAIN and nonGAIN datasets, AAO was defined as the most likely AAO of psychotic symptoms consistent with the onset of SCZ. A consensus diagnostician (PI or senior research clinician delegate) reviewed the diagnostic ratings made independently by two research diagnosticians (one of which could be the consensus diagnostician as well) and assigned a final diagnosis and AAO if the ratings were in agreement. The Kolmogorov-Smirnov test was used to determine pair-wise differences in AAO between datasets.

### GWAS and functional analyses

Quality control (QC) was conducted for each dataset separately (CIBERSAM, PsyCourse, GAIN and nonGAIN) using PLINK 1.9 [34], according to standard procedures for GWAS [35]. Briefly, genetic variants with missingness rate >2%, minor allele frequency (MAF) < 5%, Hardy-Weinberg equilibrium (HWE) *P*-value < 1e−06, and those belonging to non-autosomal chromosomes were excluded from downstream analyses. Ambiguous and multiallelic variants were also removed. Subjects with a missingness rate > 2%, increased or decreased heterozygosity rates (defined as ±3 standard deviations away from the sample mean), and relatedness >12.5% (PI_HAT > 0.125) were excluded. Sex was imputed based on X chromosome heterozygosity/homozygosity rates, before removing sex chromosomes. Principal component analyses (PCA) were conducted using SMARTPCA from EIGENSOFT 6.1.4 [36]. To keep only subjects with European ancestry, we did not use those individuals who were beyond ±3 standard deviations from the mean of the first two principal components (PCs) of the European cluster of the 1000 Genomes Project [37] Phase I. We also removed four individuals who clustered in the Finnish subgroup of the European cluster. Before genotype imputation, a PCA was performed again on the remaining subjects and the top 10 PCs were kept for further analysis.

Genotype imputation was conducted for each resulting dataset independently using the TOPMed reference panel [38]. Imputed datasets were filtered according to an imputation quality score (r-squared) <0.9 and converted to binary files using PLINK’s --vcf flag. Then, a post-imputation QC was conducted for each dataset separately, using PLINK. Only single-nucleotide polymorphisms (SNPs) were kept for further analyses. In addition, ambiguous and multiallelic SNPs, as well as SNPs with MAF < 1%, and an HWE *P*-value < 1e−06 were excluded. The resulting datasets were lifted over to genome build 19 using the UCSC liftOverPlink tool [39]. The CIBERSAM dataset included: 1704 subjects and 4,962,031 SNPs; PsyCourse: 499 subjects and 5,338,835 SNPs; GAIN: 1278 subjects and 6,042,664 SNPs; and nonGAIN: 1259 subjects and 6,074,765 SNPs. A GWAS was performed by linear regression for each dataset in PLINK, using normalized AAO as outcome and sex and the top 10 PCs as covariates. Since AAO was different between data sets a meta-analysis was then conducted using the tool METAL [40] applying an inverse variance strategy. As a result, we obtained information on 4740 subjects and 6,540,522 SNPs. As an alternative method, individual-level imputed genotype data for each of the four separate datasets was merged and a GWAS was conducted in parallel for comparison purposes. From here on, this approach is called the *merged* approach (Supplementary Note).

Genomic loci showing suggestive associations with AAO (*P*-value < 1e-05) were identified and explored using the FUMA software [41]. Each genomic risk locus was represented by the top lead SNP which had the minimum *P*-value in the locus. Lead SNPs were defined as associated SNPs (*P*-value < 1e−05) that were independent of each other at LD *r*^2^ < 0.1. Independent significant SNPs were defined as SNPs with a *P*-value < 1e−05, and independent of each other at a linkage disequilibrium (LD) threshold *r*^2^ < 0.6. The genomic risk loci were mapped to protein-coding genes by positional mapping based on ANNOVAR [42] and eQTL mapping with GTEx v8 and BRAINEAC databases. Finally, pathway enrichment analyses of the mapped genes were conducted using KOBAS-i [43]. In all the analyses, a 5% false discovery rate (FDR) was considered for multiple testing correction.

### SNP-based heritability

The proportion of phenotypic variance explained by SNPs was estimated using two different methods. First, SNP-based heritability was estimated with the Linkage Disequilibrium Score Regression (LDSC) method [44]. To reduce the standard error given our relatively small sample size, the intercept was constrained to 1 after testing that it was not significantly higher than 1 [45]. The LDSC intercept has been widely employed to distinguish between inflation due to confounding factors (such as population stratification and cryptic relatedness) and inflation due to polygenicity. Deviation of the intercept from 1 is indicative of residual confounding, thus observing an intercept not significantly higher than 1 can be interpreted as there being minimal confounding bias [44]. Second, we also estimated heritability using individual-level genotypes with the Genome-based Restricted Maximum Likelihood (GREML) approach implemented in the Genome-wide Complex Trait Analysis (GCTA) tool [46], adjusting for sex, the dataset and the top 10 PCs.

### Cross-trait polygenic risk score

PRS analyses were conducted using the PRS-cs software [47] between ten mental phenotypes and AAO. Specifically, we obtained summary statistics data on seven psychiatric disorders downloaded from the Psychiatric Genomics Consortium (https://www.med.unc.edu/pgc/) [48], including SCZ, BD, MDD, attention-deficit/hyperactivity disorder (ADHD), autism spectrum disorder (ASD), obsessive-compulsive disorder (OCD), and cannabis use disorder (CUD). In addition, we obtained GWAS data on three conditions previously associated with AAO: neuroticism [49], educational attainment (EA) [11], and childhood maltreatment [50] (Table S1). The downloaded summary statistics were filtered to remove ambiguous, multiallelic and duplicated SNPs, and SNPs with an imputation score <0.9 (if the information was provided). The summary statistics of the mental phenotypes were used as base data, and the individual-level genotype dataset was used as target data. We calculated the PRS for each mental phenotype using the “auto” mode (the shrinkage parameter phi was determined from the data with a Bayesian approach). Then, the scores obtained for each individual in our dataset were regressed out against sex, age, and batch to obtain new adjusted-PRS scores. Linear regressions were performed with the normalized AAO values as outcome and the adjusted-PRSs as independent variables to evaluate the association of each PRS with AAO. Finally, p-values were corrected for multiple correction using FDR.

### Copy number variation analysis

CNV analyses were conducted using signal intensity data from those individuals in the four cohorts from whom we obtained the intensity files (*N* = 4630). The raw CNVs were obtained using PennCNV [51]. Briefly, quality control of CNV calls was based on a sample-level criterion that examined the relationship between the standard deviation of the logarithm R Ratio (LRR_SD) and the number of CNV calls (NumCNV). At the end of the process, adjacent calls were merged together into one single call. Thresholds were carefully chosen to include as many subjects as possible but reduce false positives. Thus, subjects with LRR_SD > 0.35, BAF > 0.01, WF > 0.05, and NumCNV > 150 were not included. With all the identified CNVs we performed linear regression analyses to test whether the AAO was associated with either the number or the total length of CNVs, deletions or duplications. Sex, batch and the 10 first PCs were used as covariables. In parallel, 12 CNVs previously described as significantly associated with SCZ (SCZ-CNV) were obtained from the literature [52]. The BEDTools intersect [53] was used to look for overlaps between the described SCZ-CNVs and our data. Briefly, we selected as SCZ-CNV carriers only those individuals for whom at least 90% of the SCZ-CNV overlapped with a detected CNV (-f 0.9) and/or with at least 80% of reciprocal overlap (-r -f 0.8). To determine whether the presence of SCZ-CNVs could be influencing AAO, Wilcoxon and Kruskal–Wallis tests were performed in R4.1.2 [54], using non-normalized AAO values.

## Results

### Age at onset

Mean AAO was 23.35 (SD = 8.26). Mean AAO varied across datasets, ranging from 21.11 to 26.26 (Table 1). In the whole sample 1525 subjects (32.4%) were females (mean AAO = 25.09), and 3182 subjects (67.6%) were males (mean AAO = 22.52). Significant differences were detected between the AAO of CIBERSAM and GAIN (*P*-value < 2.2e−16), CIBERSAM and nonGAIN (*P*-value < 2.2e−16), PsyCourse and GAIN (*P*-value = 2.2e−16), and PsyCourse and nonGAIN (*P*-value = 4.44e−16). However, differences were not detected between CIBERSAM and PsyCourse (*P*-value = 0.11), and neither between GAIN and nonGAIN (*P-*value = 0.33, Fig. S1). Since the distribution of AAO was right-skewed (Fig. S1), it was normalized using a rank-based inverse-normal transformation and used in all subsequent analyses.

### GWAS and functional analyses

**Fig. 1:**
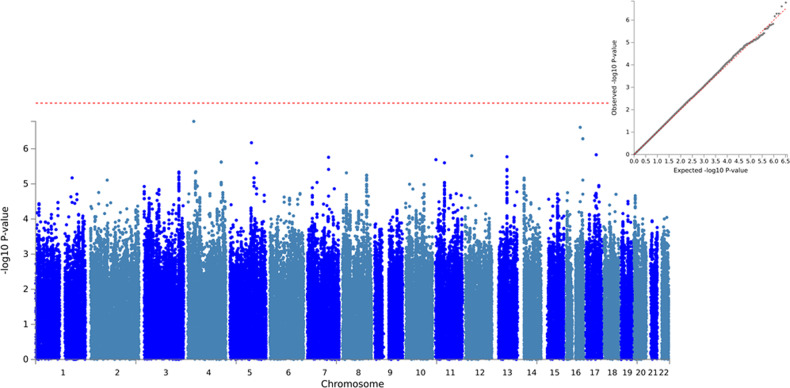
Manhattan plot (left) and Q-Q plot (upper right) of the genome-wide meta-analysis on AAO in SCZ. Red dashed line represents the threshold for genome-wide significant associations (*P*-value < 5e−08).

A total of 4740 subjects of European ancestry and 6,540,522 SNPs were included in the GWAS meta-analysis. Although none of the analyzed SNPs reached the genome-wide significant threshold (*P*-value < 5e−08, Fig. 1), 25 lead SNPs were identified, corresponding to 22 genomic risk loci that were mapped to 183 genes (Table 2). Using the Linkage Disequilibrium Score Regression (LDSC) method [44], an intercept of 1.03 (SE = 0.0083) was obtained. Mapped genes were enriched in categories such as transport of small molecules (FDR-adj *P*-value = 1.7e−02), vesicle-mediated transport (FDR-adj *P*-value = 1.8e−02), metabolism (FDR-adj *P*-value = 2.55e−02), MHC class II antigen presentation (FDR-adj P-value = 2.6e−02), and Asparagine N-linked glycosylation (FDR-adj *P*-value = 3.3e−02), among others (Table S2).

**Table 2:** Genomic risk loci for AAO identified in the genome-wide meta-analysis.

Genomic Locus	Position^a^	Lead SNP	Lead SNP position	*P*-value	nSNPs^b^	Genes
1	1:181854925	rs185188889	1:181854925	6.735e−06	1	*–*
2	2:81911239:82893571	rs13383639	82802994	7.8040E−06	19	*REG3G, REG1B, REG3A, CTNNA2, LRRTM1, SUCLG1*
3	3:173494302:173563936	rs7652242	173553275	4.57E−06	59	*NLGN1, NAALADL2*
4	4:30196116:30196116	rs111289733	30196116	1.66E−07	1	*RP11-180C1.1*
5	4:38573143:38604331	rs4833071	38582859	4.79E−06	12	*RELL1, TBC1D1, PTTG2, AC021860.1, KLF3, TLR10, RFC1, UGDH*
6	4:40443966:40443966	rs10755175	40443966	4.49E−06	1	*RHOH, RBM47, NSUN7*
7	4:167064039:167096817	rs10018884	167092502	2.36E−06	65	*MARCH1, MSMO1, SPOCK3, ANXA10, DDX60, PALLD*
8	5:108260989:108520863	rs78438786	108260989	6.72E−07	6	*FER, PJA2, MAN2A1, TMEM232, SLC25A46*
9	5:120718453:120901978	rs2195409	120892594	6.73E−06	34	*DMXL1, HSD17B4, FAM170A, FTMT, SRFBP1, LOX, SNCAIP*
10	5:133457000:133774168	rs4431386	133557876	2.54E−06	65	*SLC22A5, C5orf56, IL4, KIF3A, CCNI2, GDF9, UQCRQ, LEAP2, AFF4, ZCCHC10, HSPA4, C5orf15, VDAC1, TCF7, SKP1, CTD-2410N18.5, PPP2CA, CDKL3, UBE2B, CDKN2AIPNL, JADE2, SAR1B, SEC24A, DDX46, C5orf24, TXNDC15, PCBD2, PITX1*
11	7:48751259:48840965	rs139864446	48831950	9.07E−06	7	*AC004899.1, VWC2, C7orf72, IKZF1, DDC*
12	7:106206611:106399401	rs111513327	106207969	1.75E−06	7	*EFCAB10, ATXN7L1, SYPL1, NAMPT, CCDC71L, PIK3CG, PRKAR2B, HBP1, COG5, DUS4L, BCAP29, SLC26A4, CBLL1, SLC26A3*
13	8:20289797:20370404	rs12550821	20315601	4.85E−06	32	*CSGALNACT1, LPL, SLC18A1, ATP6V1B2, LZTS1, GFRA2, DOK2, XPO7, LGI3, SFTPC, BMP1*
14	8:121006828:121262332	rs10808509	121227216	5.61E−06	77	*TAF2, DSCC1, DEPTOR, COL14A1, MRPL13, MTBP, SNTB1*
15	11:2192798:2192798	rs10840489	2192798	2.04E−06	1	*KRTAP5-4, KRTAP5-5, KRTAP5-6, IFITM10, RP11-295K3.1, CTSD, SYT8, TNNI2, LSP1, C11orf89, TNNT3, TH, C11orf21*
16	11:44644743:44870058	rs11038082	44644743	2.51E−06	28	*C11orf96, EXT2, ALX4, CD82, TSPAN18, TP53I11, PRDM11, SYT13, SLC35C1, CRY2*
17	12:32500207:32537185	rs144642024	32500207	1.58E−06	2	*OVCH1, FAM60A, AC024940.1, DENND5B, METTL20, AMN1, KIAA1551, FGD4, DNM1L, YARS2, ALG10*
18	13:61356260:61390956	rs9570366	61377708	1.69E−06	19	*PCDH20*
19	14:20479798:20546983	rs72667672	20492904	6.82E−06	82	*OR4N2, OR4K2, OR4K5, OR4K1, OR4K14, OR4K13, OR4L1, OR4K17, OR4N5, TTC5, RNASE9, RNASE4, ANG, AL163636.6*
20	16:71388416:71388416	rs142248381	71388416	2.45E−07	1	*COG4, SF3B3, MTSS1L, VAC14, HYDIN, CMTR2, ZNF23, ZNF19, TAT, HP, HPR*
21	16:84318421:84322649	rs11645140	84322546	5.17E−07	4	*NECAB2, MBTPS1, HSDL1, DNAAF1, KCNG4, WFDC1, ATP2C2*
22	17:51482920:52487345	rs146709267	52065109	1.48E−06	11	*MBTD1, UTP18, CA10, AC102948.2, C17orf112, KIF2B, TOM1L1, COX11, STXBP4, HLF, MMD, ANKFN1, NOG*

^a^Positions are based on Human Genome version 19 (hg19), build 37.

^b^Number of SNPs in the genomic locus (*r*^2^ ≥ 0.6 with any of the independent significant SNPs).

### SNP-based heritability

The SNP-based heritability (h^2^_SNP_) was estimated using two methods that showed consistent results with moderate and significant SNP-based heritability. First, with LDSC, we obtained h^2^_SNP_ = 0.21 (SE = 0.07). SNP-based heritability was also estimated using individual-level genotypes with GCTA-GREML, adjusting for sex, dataset and the top 10 PCs, resulting in an estimate of h^2^_SNP_ = 0.17 (SE = 0.06; *P*-value = 3.33e−03). These results were consistent with the heritability estimates obtained from the GWAS *merged* approach (h^2^_SNP_ = 0.13, Supplementary Note).

### Cross-trait polygenic risk score

**Fig. 2:**
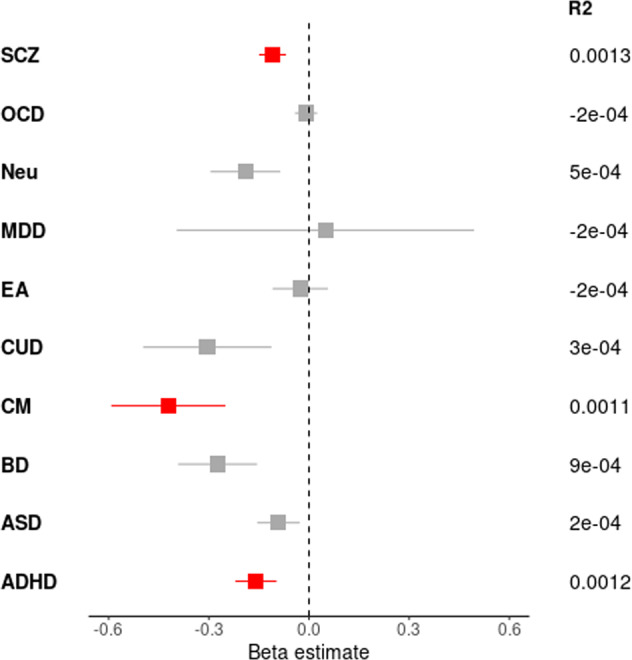
Results of the associations of adjusted-PRSs with AAO. Beta estimates (x-axis) for the adjusted-PRS of the linear model are shown. Non-significant values are colored in grey (FDR > 0.05) and significant results (FDR < 0.05) in red. Adjusted R^2^ values of the linear models are shown for each phenotype on the right side of the panel. SCZ Schizophrenia, OCD obsessive-compulsive disorder, Neu Neuroticism, MDD major depressive disorder, EA educational attainment, CUD Cannabis use disorder, CM childhood maltreatment, BD bipolar disorder, ASD Autism spectrum disorder, ADHD attention-deficit/hyperactivity disorder.

Ten mental phenotypes were examined through a cross-trait PRS analysis. Only the PRSs calculated based on ADHD, SCZ and childhood maltreatment sumstats were associated with AAO in our dataset (FDR < 0.05). All adjusted PRS beta coefficients were negative, corresponding to the higher burden of disease/condition risk variants being associated with earlier onset of SCZ (Fig. 2 and Table S3). The variance explained by the adjusted-PRS of these three phenotypes was low but significant (1.2e−03; 1.3e−03 and 1.1e−03, respectively for ADHD, SCZ, and childhood maltreatment). Among the other mental phenotypes, only BD-PRS was nominally associated with AAO (*P* = 0.02).

### CNV analysis

After quality control, a total of 3965 individuals remained for CNV downstream analyses and a total of 107,668 deletions (mean number = 27.18 ± 17.12; mean length = 1434 kb ± 1617 kb) and 59,336 duplications (mean number = 15.05 ± 10.09; mean length = 2094 kb ± 6817 kb) were identified. 3962 individuals (99.92%) carried at least one deletion and 3942 (99.41%) individuals carried at least one duplication. AAO was significantly associated with the total number of CNVs (beta = −0.015; *P*-value = 0.025), and also with the number of deletions (beta = −0.02; *P*-value = 0.03) and the length of the deletions (beta = −1.8e−7; *P*-value = 0.03, Table 3). Out of 3965, 117 individuals (3%) carried SCZ-CNVs previously associated with SCZ, and 4 individuals carried two SCZ-CNVs. Among the SCZ-CNV-carriers, we detected 36 (29.8%) carrying 15q11.2del, 20 (16.53%) 22q11.2del, 13 (10.74%) 16p12.1del, 12 (9.92%) 16p11.2dup, 11 (9.09%) 16p13.11dup, 10 (8.26%) 15q13.3del, 6 (4.96%) 3q29del, 4 (3.31%) carrying 1q21.1dup, 4 (3.31%) 15q11-q13dup, 3 (2.48%) 1q21.1del, and 2 (1.65%) 7q11.23dup individuals. The deletion corresponding to 2p16.3 was not present in our sample. In our dataset, the presence of SCZ-CNVs was not associated with an earlier AAO (Wilcoxon test; *P*-value = 0.73, Fig. S2A). In addition, no differences in the AAO were found across SCZ-CNVs (Kruskal–Wallis test; *P*-value = 0.49, Fig. S2B).

**Table 3:** Results from linear models for length and number of CNVs, deletions and duplications.

	Number of CNVs		Number of deletions		Number of duplications	
	Beta	*P*	Beta	*P*	Beta	*P*
Number	−0.015	**0.025**	−0.017	**0.029**	0.009	0.492
PC1	−15.50	0.054	−15.947	**0.046**	−15.786	**0.048**
PC2	−8.43	0.293	−9.760	0.221	−9.682	0.225
PC3	1.91	0.813	0.643	0.936	1.295	0.872
PC4	−3.43	0.673	−1.059	0.896	0.593	0.941
PC5	5.07	0.523	4.505	0.568	3.521	0.655
PC6	7.17	0.366	5.659	0.473	5.314	0.501
PC7	−3.21	0.683	−2.753	0.725	−3.220	0.681
PC8	9.09	0.249	9.584	0.221	9.817	0.211
PC9	1.45	0.853	1.507	0.846	1.499	0.847
PC10	−5.64	0.476	−6.727	0.392	−6.771	0.389
Sex	2.43	**<2e−16**	−2.406	**<2e−16**	2.382	**<2e−16**
Batch	−1.24	**<2e−16**	−3.265	**<2e-16**	−3.550	**<2e−16**
adj-R^2^	0.065		0.075		0.075	
Model pval	<2.2e^−16^		<2.2e^−16^		<2.2e^−16^	

	Length of CNVs		Length of deletions		Length of duplications	
	Beta	*P*	Beta	*P*	Beta	*P*
Length	-4.77E-09	0.798	0.000	**0.029**	0.000	0.809
PC1	−15.74	**0.049**	−0.160	**0.045**	−0.157	**0.049**
PC2	−9.63	0.228	−9.650	0.226	−9.693	0.225
PC3	1.07	0.894	1.480	0.854	1.225	0.879
PC4	0.60	0.941	−0.010	0.990	0.441	0.956
PC5	3.59	0.649	4.010	0.610	3.618	0.646
PC6	5.26	0.505	5.810	0.462	5.289	0.503
PC7	−3.16	0.686	−3.015	0.700	−3.181	0.684
PC8	9.88	0.208	10.050	0.200	9.688	0.217
PC9	1.51	0.846	1.760	0.821	1.482	0.849
PC10	−6.77	0.390	−6.745	0.391	−6.779	0.389
Sex	2.39	**<2e−16**	2.490	**<2e−16**	2.380	**<2e−16**
Batch	−3.47	**<2e−16**	−3.250	**<2e−16**	−3.522	**<2e−16**
adj-R^2^	0.074		0.075		0.074	
Model pval	<2.2e^−16^		<2.2e^−16^		<2.2e^−16^	

Bold values identify statistical significance (*p* < 0.05).

## Discussion

This study explored the genetic architecture of AAO of SCZ. To this end, we performed a case-only GWAS of European ancestry in the largest sample collected to date. Although no genome-wide significant signals were detected, we successfully estimated for the first time the SNP-based heritability of AAO using two different approaches that showed consistent results of moderate heritability, ranging from 17 to 21%. The fact that we did not identify any specific genetic variants associated with AAO suggests that the heritability of the trait is likely to be complex, involving multiple genetic and environmental factors. We also provided evidence of negative genetic associations of cross-trait PRS derived from ADHD, SCZ, and childhood maltreatment with AAO. Finally, we determined that the burden of deletions was associated with AAO in our dataset.

In our study, the strongest association signal was found in a genomic locus at chromosome 4 (lead SNP rs111289733 *P*-value = 1.66e−07), which harbored the long non-coding RNA *RP11-180C1.1*. The mapped genes belonging to the suggestive associations were enriched in transport of small molecules, vesicle-mediated transport, Asparagine N-linked glycosylation, and MHC class II antigen presentation, among others. Both the transport of molecules and, specifically, the vesicular transport mechanism might participate in the pathogenesis of SCZ by triggering dysfunctional neuroexocytosis [55, 56]. In fact, previous studies reported abnormal reductions in synaptic vesicle proteins being associated with SCZ [57–59]. Also, MHC has been strongly associated with the risk of SCZ [60]. In addition, glycosylation involves processes critical for normal brain development and has been suggested to contribute to the abnormal neuronal signaling and connectivity observed in SCZ [61]. Altogether, these categories are promising candidates for further studies on pathways associated with AAO.

Over the years, many studies have evaluated the role of genetics in AAO of SCZ [62, 63], estimating a heritability of AAO ranging from approximately 20 to 58% [7]. In our study, based on two different methodologies we estimated the SNP-based heritability of AAO to be between 17 and 21%. In fact, our estimates show consistent results suggesting a moderate but significant contribution of common variants to AAO. Interestingly, SNP-based heritability is slightly higher than that of AAO in BD and MDD, which has recently been estimated at 5 and 6% respectively, using larger sample sizes [27]. Further studies with larger sample sizes are needed to obtain more accurate estimates in SCZ AAO.

Cross-trait PRSs constructed with ADHD, SCZ, and childhood maltreatment were significantly associated with AAO, showing that a higher risk of developing these conditions is associated with an earlier AAO in SCZ. However, they explained a very small fraction of AAO variation [64]. Previous studies reported that ADHD was among the commonest comorbidities in children and adolescents with SCZ [65], and it has been argued that the genetic architecture of ADHD has a large link with SCZ [66]. In this line, our results suggest that an earlier AAO may be related to a more severe neurodevelopmental impairment. AAO has been suggested as a potential endophenotype of SCZ, reflecting the underlying genetic architecture of the disorder. Our finding that the PRS of SCZ can predict AAO supports this notion and suggests that AAO could be a useful predictor of disease severity. It has also been suggested that individuals with higher genetic loadings for SCZ are at a higher risk of early onset [67], and similarly for MDD [28]. Moreover, patients with histories of being abused as children show an earlier onset of symptoms [68]. Here, we also report a negative association between AAO and the PRS of both SCZ and childhood maltreatment. However, it is still unknown how the genetic architectures of these traits are linked.

A recent study reported that the prevalence of recurrent CNVs was higher in early onset psychosis than in the general population, as well as CNV pathogenicity [67]. In addition, some of these CNVs cause earlier-onset disorders such as developmental delay or ASD, but not SCZ [20]. Here, we observed that the burden of CNVs, especially deletions (either the length or the total number), but not duplications, were associated with an earlier onset of SCZ, suggesting a combined role of common variation and CNVs in determining AAO. However, in our study, neither the presence of pathogenic SCZ-CNV, nor any specific SCZ-CNVs were associated with AAO. Nevertheless, we detected a 1.9% prevalence of the pathogenic SCZ-CNVs, which is close to the previously reported prevalence of 2.6% [20].

Our study has some strengths and limitations that deserve discussion. Despite the lack of genome-wide significant associations at the SNP-level, which could be expected given the relatively small sample size of our study, we were able to estimate the SNP-based heritability of AAO and identify relevant associations with the PRS of three mental phenotypes for the first time. This finding represents an important step toward elucidating the genetic architecture of AAO. It is worth noting that studying AAO is both technically and conceptually challenging, as there is often considerable variability in how AAO is defined and measured between studies. Although these measures have been shown to be correlated and to occur within a relatively short time frame [6, 69], it is possible that our results may be confounded by different definitions of AAO between datasets. In our study, we acknowledge that AAO differed between the Europe and US cohorts in our study and therefore performed a GWAS meta-analysis to minimize any potential bias due to phenotype heterogeneity. However, the fact that AAO did not differ within the European (CIBERSAM and PsyCourse) and the US (GAIN and nonGAIN) cohorts suggests that the phenotypes were obtained in a homogeneous manner. In the future, more reliable estimates of AAO may lead to the identification of significant signals and an increase in the heritability explained by SNPs. Also, the rank-based transformation applied to the AAO values may have affected our GWAS and subsequent analyses; however, it is considered as one of the best approaches to use. Some studies have reported that for small sample sizes or genetic effects, there is an improvement in sensitivity for rank-based transformations that outweighs a slight increase in the false-positive rate [70]. In addition, a recent study has demonstrated that these transformation tests outperform the standard untransformed association test, both in terms of power and type I error rate control [71]. Moreover, we were not able to control for putative differences between the different recruitment sites. All the datasets included in the study might be multicenter; thus, heterogeneity within datasets could be considerable. Such phenotypic heterogeneity has been reported to affect genetic analyses [27, 72], which indicates that phenotype harmonization is as important as a larger sample size for improving the power to detect significant associations and avoiding a biased view of genetic architectures.

## Conclusions

In conclusion, we report on the largest GWAS of AAO in SCZ to date, providing the first SNP-based heritability estimate of AAO in individuals of European ancestry. Although no genome-wide significant SNP was detected, we provide evidence of a genetic background for AAO and a negative association with the PRS of ADHD, SCZ and childhood maltreatment. In addition, we demonstrate that the burden of deletions is associated with the AAO of the disease. Larger sample sizes are needed to fully determine the genetic architecture of AAO, which could help us understand further the pathogenesis of SCZ and contribute to the development of better strategies for the early detection of SCZ. Nonetheless, our study provides an important step forward in understanding the genetic architecture of AAO.

### Supplementary information

Supplementary Note

Supplementary Tables

## Data Availability

Given the collaborative nature of the CIBERSAM cohort and the involvement of multiple institutions and research groups, requests for access to the genotype data should be addressed to the corresponding author [muntaneg@peremata.com] to initiate the data access request. Acces to Psycourse dataset can be requested thorugh a “Secondary Analysis Proposal” at http://www.psycourse.de/openscience-en.html.
