# Correction: Common genetic variants contribute to heritability of age at onset of schizophrenia

**DOI:** 10.1038/s41398-023-02651-8

**Published:** 2023-11-30

**Authors:** Ester Sada-Fuente, Selena Aranda, Sergi Papiol, Urs Heilbronner, María Dolores Moltó, Eduardo J. Aguilar, Javier González-Peñas, Álvaro Andreu-Bernabeu, Celso Arango, Benedicto Crespo-Facorro, Ana González-Pinto, Lourdes Fañanás, Barbara Arias, Julio Bobes, Javier Costas, Lourdes Martorell, Thomas G. Schulze, Janos L. Kalman, Elisabet Vilella, Gerard Muntané

**Affiliations:** 1 Department of Psychiatry, Hospital Universitari Institut Pere Mata, Institut d’Investigació Sanitària Pere Virgili (IISPV), Universitat Rovira i Virgili (URV), Reus, Spain; 2 Centro de Investigación Biomédica en Red de Salud Mental (CIBERSAM), Madrid, Spain; 3 Institute of Psychiatric Phenomics and Genomics (IPPG), University Hospital, Ludwig Maximilian University of Munich, 80336 Munich, Germany; 4 Department of Psychiatry and Psychotherapy, University Hospital, Ludwig Maximilian University of Munich, 80336 Munich, Germany; 5 Department of Genetics, Universitat de Valencia, 46100 Valencia, Spain; 6 Biomedical Research Institute INCLIVA, 46010 Valencia, Spain; 7 Department of Psychiatry, Hospital Clínico Universitario de Valencia, 46010 Valencia, Spain; 8 Faculty of Medicine, Universidad de Valencia, 46010 Valencia, Spain; 9 Department of Child and Adolescent Psychiatry, Institute of Psychiatry and Mental Health, Gregorio Marañón Health Research Institute (IiSGM), Hospital General Universitario Gregorio Marañón, 28007 Madrid, Spain; 10 Faculty of Medicine, Universidad Complutense, 28007 Madrid, Spain; 11 Department of Psychiatry, Universidad de Cantabria, 39005 Santander, Cantabria Spain; 12 Hospital Universitario Marqués de Valdecilla-IDIVAL, 39008 Santander, Cantabria Spain; 13 Department of Psychiatry, University Hospital Virgen del Rocío, Instituto de Biomedicina de Sevilla (IBiS), Sevilla, Spain; 14 Department of Psychiatry, Hospital Universitario Araba, Instituto de Investigación Sanitaria Bioaraba, Universidad del País Vasco, 01009 Vitoria, Spain; 15 Department of Evolutionary Biology, Ecology and Environmental Sciences, Faculty of Biology, Universitat de Barcelona, Institut de Biomedicina de la Universitat de Barcelona (IBUB), 08028 Barcelona, Spain; 16 Faculty of Medicine and Health Sciences - Psychiatry, Universidad de Oviedo, 33006 Oviedo, Spain; 17 Mental Health Services of Principado de Asturias (SESPA), Instituto de Investigación Sanitaria del Principado de Asturias (ISPA), 33011 Oviedo, Spain; 18 Instituto de Neurociencias del Principado de Asturias (INEUROPA), 33003 Oviedo, Spain; 19 Psychiatric Genetics Group, Instituto de Investigación Sanitaria de Santiago de Compostela (IDIS), Servizo Galego de Saúde (SERGAS), Complexo Hospitalario Universitario de Santiago de Compostela (CHUS), 15706 Santiago de Compostela, Spain; 20 Department of Psychiatry and Behavioral Sciences, SUNY Upstate Medical University, Syracuse, NY USA; 21 Institut de Biologia Evolutiva (UPF-CSIC), Departament de Medicina i Ciències de la Vida, Universitat Pompeu Fabra, Parc de Recerca Biomèdica de Barcelona, Barcelona, Spain

**Keywords:** Schizophrenia, Genomics

Correction to: *Translational Psychiatry* 13, 201 (2023); 10.1038/s41398-023-02508-0, published online 13 June 2023

The Acknowledgements section in this Article was incomplete.

“This work was supported by Instituto de Salud Carlos III (PI18/00514 and PI21/00612) and by the Catalan Agency of Research and Universities (AGAUR, 2017SGR-00444 and 2021SGR01065). The PsyCourse study was supported by DFG (SCHU 1603/4-1, 5-1, 7-1, FA241/16-1).”

should read:

“This work was supported by Instituto de Salud Carlos III through the projects PI18/00514 and PI21/00612 and co-funded by the European Union, by the Catalan Agency of Research and Universities (AGAUR, 2017SGR-00444 and 2021SGR01065). The PsyCourse study was supported by DFG (SCHU 1603/4-1, 5-1, 7-1, FA241/16-1)."

The original article has been corrected.

