# Supplementary material for: Common genetic variants contribute to heritability of age at onset of schizophrenia
Source: Transl Psychiatry. 2023 Jun 13;13:201. doi: 10.1038/s41398-023-02508-0 (PMC10261125; doi:10.1038/s41398-023-02508-0)
Supplement: Supplementary file 1 — Supplementary Note [file 41398_2023_2508_MOESM1_ESM.docx]

**SUPPLEMENTARY NOTE**

1. **Individual-level combined GWAS as an alternative approach**

Quality control (QC) was conducted according to standard procedures for GWAS before the genetic association analysis ^1^. First, QC was performed separately for each dataset using PLINK 1.90 ^2^. Briefly, genetic variants with missingness rate > 2%, minor allele frequency (MAF) < 5%, Hardy-Weinberg equilibrium (HWE) P-value < 1e-06, and those belonging to non-autosomal chromosomes were excluded from downstream analyses. Ambiguous and multiallelic variants were also removed. Subjects with missingness rate > 2%, increased or decreased heterozygosity rates (defined as ±3 standard deviations away from the sample mean), and relatedness > 12.5% (PI_HAT > 0.125) were excluded. Sex was imputed based on X chromosome heterozygosity/homozygosity rates, before removing sex chromosomes from the analyses. After QC steps, principal component analyses (PCA) were conducted using SMARTPCA from EIGENSOFT 6.1.4 ^3^. To keep only subjects with European ancestry, we discarded those individuals beyond ±3 standard deviations from the first two principal components (PCs) mean of the European cluster of 1000 Genomes Project Phase I ^4^. At this point, binary files of the four datasets were temporarily merged. In this merged dataset, we evaluated relatedness between individuals and one subject from pairs of related subjects of the GAIN and nonGAIN studies (N = 37) were excluded. Subsequently, a PCA was performed again on the remaining subjects and the top 10 PCs were kept for further analysis.

Genotype imputation was conducted independently for each dataset (CIBERSAM, PsyCourse, GAIN, and nonGAIN) using the TOPMed Imputation server ^5^. Imputed datasets were filtered discarding genetic variants with an imputation quality score (r-squared) < 0.9, and converted to binary files using PLINK’s --vcf flag. Finally, the four datasets were merged, post-imputation QC was applied using PLINK (ambiguous and multiallelic SNPs, as well as SNPs with MAF < 1%, and HWE P-value < 1e-06 were excluded), and the genotypes were lifted over to genome build 19 using the UCSC liftOverPlink tool ^6^. At the end of the process, the resulting dataset included 4 707 individuals and 4 649 950 SNPs. GWAS was performed by linear regression in PLINK using normalized AAO as the outcome and sex, dataset, and the top 10 PCs were included as covariates.

**Results**

Genomic loci showing suggestive associations with AAO (*P*-value < 1e-05) were identified using FUMA ^7^. Although none of the SNPs reached the genome-wide significance threshold (*P*-value < 5e-08), we detected 33 suggestive associations (*P*-value < 1e-05) (Figure S3, Table S4). The three strongest association signals for AAO were intergenic variants at chromosomes 16 (rs142248381, *P*-value = 6.093e-07, nearest gene *CALB2*), 13 (rs9570366, *P*-value = 1.404e-06, nearest gene *RNY4P31*) and 8 (rs12550821, *P*-value = 1.425e-06, nearest genomic element was a long non-coding RNA *RP11-563N12.2*). Thirteen lead SNPs were detected, corresponding to 12 genomic risk loci, which were mapped to 98 genes (Table S4). No gene showed a significant association with AAO (5% FDR) in the gene-based analysis. The LDSC analysis estimated an intercept of 1.01 (SE = 0.0081), consistent with minimal population stratification or cryptic relatedness (λ_GC_ = 1.003).

**SNP-based heritability**

The contribution of common variation to heritability showed results consistent with moderate and significant SNP-based heritability. With LDSC, the SNP-based heritability was estimated as h^2^_SNP_ = 0.13 (SE = 0.07).

**References**

1. Marees, A. T. *et al.* A tutorial on conducting genome-wide association studies: Quality control and statistical analysis. *Int J Methods Psychiatr Res* **27**, e1608 (2018).

2. Purcell, S. *et al.* PLINK: a tool set for whole-genome association and population-based linkage analyses. *Am J Hum Genet* **81**, 559–575 (2007).

3. Patterson, N., Price, A. L. & Reich, D. Population Structure and Eigenanalysis. *PLoS Genet* **2**, e190 (2006).

4. 1000 Genomes Project Consortium *et al.* A global reference for human genetic variation. *Nature* **526**, 68–74 (2015).

5. NHLBI Trans-Omics for Precision Medicine (TOPMed) Consortium *et al.* Sequencing of 53,831 diverse genomes from the NHLBI TOPMed Program. *Nature* **590**, 290–299 (2021).

6. Navarro Gonzalez, J. *et al.* The UCSC Genome Browser database: 2021 update. *Nucleic Acids Res* **49**, D1046–D1057 (2021).

7. Watanabe, K., Taskesen, E., Van Bochoven, A. & Posthuma, D. Functional mapping and annotation of genetic associations with FUMA. *Nature Communications* **8**, 1–11 (2017).

**SUPPLEMENTARY FIGURES**

| **A)**  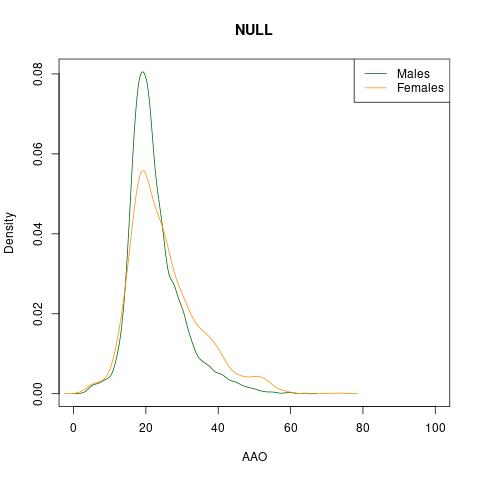 | 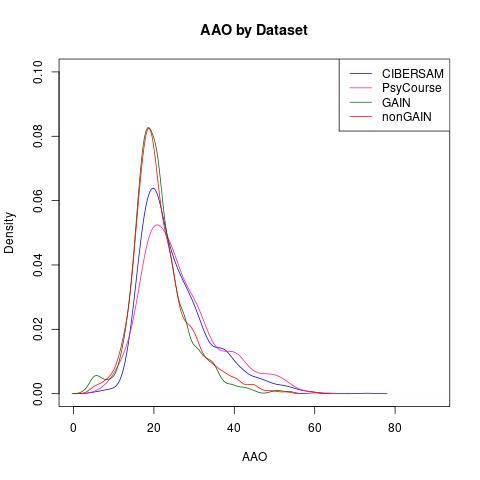**B)** | **C)**  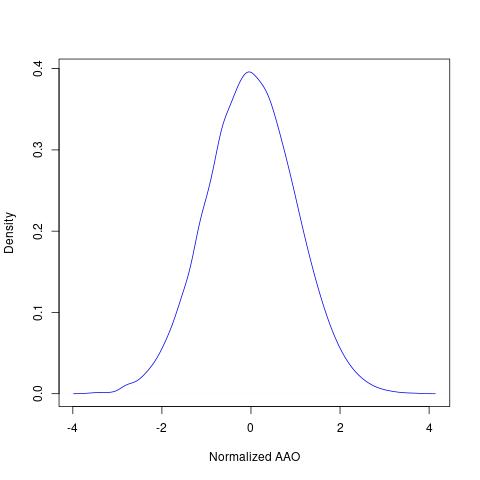 |
| --- | --- | --- |
| **Figure S1.** A) Distribution of AAO based on sex. Mean AAO in males was 22.52, while mean AAO in females was 25.09. B) Distribution of AAO for each dataset. C) Distribution of normalized AAO by rank-based inverse-normal transformation. | | |

| **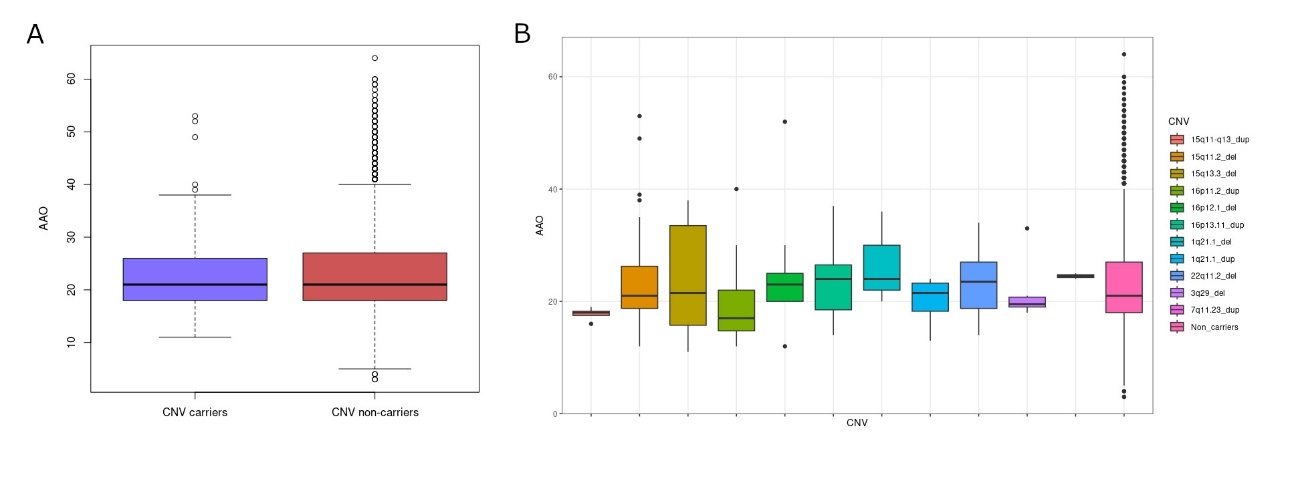** |
| --- |
| **Figure S2.** A) Comparison of AAO between carriers and non-carriers of the 12 CNVs most significantly associated with SCZ. No differences were found (Wilcoxon test, P-value = 0.85). B) Comparison of AAO among carriers and non-carriers of each of the 11 CNVs previously associated with SCZ. No differences were found (Kruskal-Wallis test, P-value=0.73). |

| 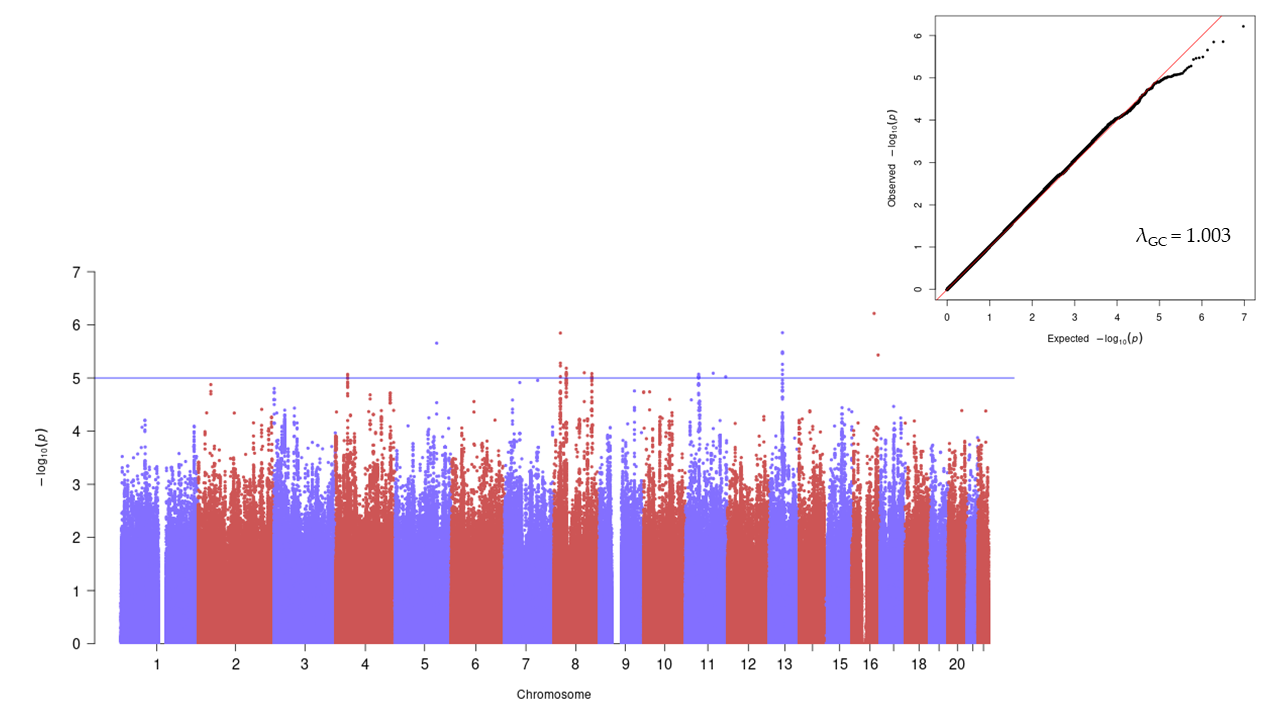 |
| --- |
| **Figure S3.** Manhattan plot (left) and Q-Q plot (upper right) of the GWAS results from the *merged* approach on AAO in SCZ in a combined sample of European ancestry. The blue line represents the threshold for suggestive associations (P-value < 1e-05). |
